# Low carbohydrate diets, glycaemic control, enablers, and barriers in the management of type 1 diabetes: a mixed methods systematic review

**DOI:** 10.1186/s13098-024-01496-5

**Published:** 2024-11-02

**Authors:** Janine Paul, Rati Jani, Sarah Thorning, Mila Obucina, Peter Davoren, Catherine Knight-Agarwal

**Affiliations:** 1 School of Clinical Science, Faculty of Health, University of Canberra, Bruce, ACT 2617 Australia; 2 Diabetes and Endocrinology, Adult Outpatient Department, Gold Coast University Hospital and Health Service, 1 Hospital Boulevard, D Block, Area 4, Southport, QLD 4215 Australia; 3 School of Health Sciences and Social Work, Griffith University, Southport, QLD 4215 Australia; 4 Education and Research Unit, Central Queensland Hospital and Health Service, Rockhampton, QLD 4700 Australia; 5 Emergency Department, Gold Coast University Hospital and Health Service, Southport, QLD 4215 Australia; 6 School of Medicine and Dentistry, Griffith University, Southport, QLD 4215 Australia

**Keywords:** Diabetes type 1, Very low carbohydrate diet; low carbohydrate diet; quantitative, Qualitative, Adults, Mixed methods systematic review

## Abstract

**Background:**

Medical nutrition therapy is fundamental for diabetes management, however there is a lack of evidence supporting an ideal recommended carbohydrate intake for maintaining optimal glycaemia in individuals living with type 1 diabetes (T1D). Adults with T1D are increasingly drawn to very low carbohydrate (≤ 50 g/day or < 10% total energy intake) and low carbohydrate diets (< 130 g/day or < 26% total energy intake) because of the reported positive impact on both physical health and psychological well-being. Current evidence regarding the effectiveness on glycaemia and the lived experience by adults with T1D when using these diets is limited. This mixed methods systematic review was undertaken to examine the effectiveness of very low and low carbohydrate diets on HbA1c and explore the lived experience of adults with T1D who have followed these dietary regimens.

**Methods:**

Seven databases (MEDLINE, Embase, CINAHL, Cochrane CENTRAL, Informit Health Collection, Web of Science, and PsycInfo) were searched from inception to 1 October 2023. Quality assessment of the included studies was undertaken using the JBI’s critical appraisal checklists. Separate quantitative and qualitative synthesis was performed, and findings were integrated for the purpose of comparison and complementarity.

**Results:**

Seventeen studies of varying methodologies were included. Findings from quantitative research were inconclusive in determining the effectiveness of very low and low carbohydrate diets on HbA1c levels. Qualitative data synthesis identified four themes [1) Motivation to follow the diet, 2) Health benefits of the diet, 3) Challenges of the diet, and 4) Limited information (participants knowledge, information sources) about the diet] that influenced adherence to very low and low carbohydrate diets. Through the integration of results from selected studies, it was evident that there were conflicting outcomes between quantitative and qualitative studies.

**Conclusions:**

There is little evidence to indicate that very low and low carbohydrate diets improve HbA1c in adults with T1D. However, this goes against the reported lived experiences of participants. This review highlights the insufficiency of robust evidence on this topic. Future research involving larger participant samples over longer durations are needed to provide more definitive evidence in relation to the efficacy of these diets and into the enablers and barriers experienced when using a very low or low carbohydrate diet in order to provide support to adults with T1D.

*Systematic review registration* PROSPERO CRD42023482800.

**Supplementary Information:**

The online version contains supplementary material available at 10.1186/s13098-024-01496-5.

## Background

According to the International Diabetes Federation, 537 million people aged 20 to 79 years worldwide live with diabetes [1]. Of these 5–15% have type 1 diabetes [1, 2]. Type 1 diabetes (T1D) is an autoimmune condition characterised by chronic hyperglycaemia which results in absolute endogenous insulin deficiency [3]. Achieving glycaemia (target HbA1c ≤ 7.0% or < 53 mmol/mol) is associated with optimal diabetes management [4]. Data available from T1D registries (2010–2013) for 324,501 patients across 19 countries in Europe, North America, and Australasia reported that 71% of adults’ (aged ≥ 25 years) HbA1c was > 7.0% (> 53 mmol/mol) [5]. Chronic hyperglycaemia (HbA1c > 7.0%) contributes to the development of macro- and microvascular disease [4].

Medical nutrition therapy is considered an integral aspect of diabetes management [6]. Carbohydrate is the main macronutrient that influences postprandial blood glucose [7]. To date, there is no established ideal carbohydrate intake for maintaining long-term optimal glycaemia in people living with T1D [8]. However, emerging findings, although predominantly from studies of lesser quality such as quasi experimental, case series, and case reports, indicate that adopting a very low carbohydrate diet (VLCD) (≤ 50 g/day or < 10% TEI) [9–17] or low carbohydrate diet (LCD) (< 130 g/day or < 26% TEI) [7, 18–22] may help improve HbA1c.

The adoption of VLCDs and LCDs have been investigated among people with type 2 diabetes (T2D) [6, 23]. A recent meta-analysis found that diets where carbohydrate intake constituted < 26% TEI, yielded a statistically significant reduction in HbA1c at three month [weighted mean difference = -0.47% (95%CI -0.71 to -0.23%)] in T2D adults [6]. However, there is a lack of long-term robust evidence related to the effectiveness of these diets in achieving optimal HbA1c in adults with T1D. To-date, there is only one meta-analysis that examined the efficacy and safety of reduced carbohydrate diets in adolescents and adults with T1D [24]. This study included a small sample of nine randomised control trials (RCTs) identifying no VLCD interventions (≤ 50 g/day or < 10% TEI), four LCDs (< 130 g/day or < 26% TEI), four moderate carbohydrate diets (MCD) (130-230 g/day or 26–45% TEI), and one study that used both a LCD and MCD. This meta-analysis found that the LCDs (*n* = 4) had no significant influence on HbA1c [mean difference = -0.16% (95%CI -0.67 to 0.35), *I*^2^ = 58%, *P* = 0.07] [24]. Analysis including all nine RCTs which included both LCDs and MCDs studies found no significant effect on HbA1c [mean difference = 0.01% (95%CI -0.33 to 0.35%), *I*^2^ = 39%, *P* = 0.17]. These findings could be attributed to the limited evidence searched (i.e., only two databases were screened), small sample sizes across included studies, and methodological differences in individual study designs [24].

Nevertheless, despite limited evidence on the effectiveness of VLCDs and LCDs in the management of T1D, there is a growing amount of anecdotal evidence advocating for their use. This is evidenced by an increase in media and community interest [25–27], the proliferation and popularity of LCD books [28, 29], and a burgeoning availability of low carbohydrate foods such as breads, pasta, and breakfast cereals [30]. There is also an increase in individuals with T1D who have reportedly trialed both the general population dietary guidelines (a high carbohydrate diet) and a VLCD or LCD with the latter being found to have a beneficial influence on diabetes management [11, 15, 25, 31, 32]. These individuals reported that a VLCD or LCD not only improved HbA1c levels but had a positive impact on their physical and psychological well-being [10, 11, 15, 25, 31, 32].

Individuals living with T1D endure ongoing, lifetime management of daily blood glucose monitoring, and intensive insulin treatment, with some experiencing various levels of psychological maladjustment [33, 34]. Unsurprisingly, a strong association between poor diabetes management and depressive symptoms has been observed by scholars [35, 36]. Hence, a gap in the evidence was identified regarding the lived experiences of those with T1D, who have chosen to adopt a VLCD or LCD, particularly in relation to their quality of life (QoL) and the factors that influence or hinder their dietary choice. Therefore, given the anecdotal evidence pointing to the advantages of VLCDs and LCDs, the authors sought to explore the published evidence on the broader benefits and challenges associated with these diets.

To-date, there are no new quantitative studies that have been published since the 2023 meta-analysis and there is no existing research that has taken the next step of synthesising both quantitative and qualitative studies on this topic. Our research is unique in its approach, we aim to integrate both quantitative and qualitative data to offer a comprehensive understanding of the effects of LCDs, glycaemia, enablers, and barriers in the care of T1D. Therefore, this study aims to address this gap, and 1) examine the effectiveness (quantitative evidence) of a VLCD or LCD on HbA1c in adults living with T1D and 2) explore the perceptions, knowledge, and experience (qualitative evidence) of adults living with T1D who have used these dietary regimens, by means of a mixed methods systematic review.

## Methods

### Protocol

The Preferred Reporting Items for Systematic Reviews and Meta-Analyses (PRISMA) guidelines have been used to report this reviews’ findings [37] (Additional file 1). This review was registered with PROSPERO (registration number: CRD42023482800).

### Search strategy

Seven scientific databases were searched including MEDLINE, Embase, CINAHL, Cochrane CENTRAL, Informit Health Collection, Web of Science, and PsycInfo to identify published studies on the topic of interest from inception to 1 October 2023. The search strategy included subject headings and keywords for terms such as “diabetes mellitus, type 1”, “insulin dependent” combined with diet related search terms such as “diet”, “carbohydrate-restricted”, “keto”, and linked using Boolean phrases. The full search strategy developed for the MEDLINE (Ovid) database is shown in Additional file 2. Reference lists of all included manuscripts were hand searched for additional potentially relevant studies. This review followed the Joanna Briggs Institute Mixed Methods Systematic Review protocol to ensure a comprehensive and structured analysis of the research topic [38]. A convergent integrated approach was used to synthesize the current evidence by combining findings on the effectiveness (quantitative evidence) and perceptions, knowledge, and experience (qualitative evidence) of adults with T1D who have adopted a VLCD or LCD. The primary quantitative outcome for this review will be HbA1c (pre- to post-intervention) and the secondary outcomes will include bolus insulin, weight, and QoL of included studies. In addition, the perceptions, knowledge, and experiences of adults with T1D who have adopted a VLCD or LCD will be explored with outcomes being conveyed through qualitative studies.

### Inclusion and exclusion criteria

#### Population

The population for this review included adults (aged ≥ 18 years) of all genders and ethnic backgrounds who held a diagnosis of T1D for ≥ one year. Studies were excluded if participants: (1) were aged ≤ 17 years, (2) had T2D, (3) had gestational diabetes, (4) were breast-feeding, and (5) were non-human.

#### Phenomenon of interest

This review included studies that investigated the use of a VLCD (≤ 50 g/day or < 10% TEI) or LCD (< 130 g/day or < 26% TEI) [39]. The carbohydrate dietary intervention could be combined with a high protein or high fat intake, or both. Qualitative studies were included where participants followed a VLCD or LCD and their perceptions, knowledge, and experiences were reported.

#### Context

This review included studies where participants undertook a VLCD or LCD in their natural setting.

#### Types of study

This review considered only primary studies i.e., quantitative, qualitative, and mixed methods studies published in English. All study designs (i.e., experimental/interventional and non-experimental/observational) regardless of study duration or sample size were considered eligible, including qualitative studies applying various methodologies and mixed studies employing diverse approaches. Studies that reported changes glycated haemoglobin (HbA1c) (pre to post study) were included. Studies that only reported pre or post HbA1c were excluded. Published commentaries, secondary studies (such as reviews and meta-analysis), editorials, conference papers, book chapters, discussion papers with no original data, thesis, and grey literature were excluded. Moreover, studies with both T1D and T2D and children/adolescents and adult populations were included, if the results of each group were clearly reported. Studies where this distinction was lacking were excluded. An in-depth overview of the inclusion and exclusion criteria for both quantitative and qualitative studies is shown in Additional file 3.

### Study screening and selection

Search results were imported to Endnote 20 and duplicates removed. Covidence software was used to guide the screening and data extraction process [40]. Data extraction forms were pilot tested, and screening was completed in duplicate for two of each type of study design. Three researchers (JP, ST, CKA) independently reviewed the abstracts for adherence to the selection criteria. This ensured consensus between the authors to avoid misunderstanding or later disagreement prior to conducting the full data extraction process [41]. Inter-rater reliability agreement was assessed using Cohen’s kappa (*k*) [41]. The values are interpreted as follows: < 0.00 as no agreement, 0.00–0.20 as slight, 0.21–0.40 as fair, 0.41–0.60 as moderate, 0.61–0.80 as substantial, and 0.81–1.00 as perfect agreement [41]. Following pilot testing and the duplicate screening process, JP, ST, and CKA independently performed titles and abstract screening, followed by full-text article appraisal of the remaining studies. Any discrepancies between the reviewers were discussed. A fourth reviewer (MO) was consulted when consensus could not be reached.

### Data extraction

The review team developed a purpose-built data extraction template to capture relevant information from individual studies. For quantitative studies, details included: (1) author/year/country of origin, (2) study characteristics, (3) participant characteristics, and (4) selected outcomes. For qualitative studies, details recorded included: (1) author/year/country of origin, (2) research aim/objectives, (3) participant characteristics, (4) study design/study setting, (5) intervention duration/year of data collection, and (6) key findings/conclusions [42]. Additional file 4 summaries the key variables extracted from included quantitative and qualitative studies.

Furthermore, the data extracted from each outcome was reported as either a mean ± SD, percentage, or median. Where data such as SD was missing, we contacted the author for required information. Where authors did not respond to our request for data then the published findings were reported and the missing data; NR (not reported) was used to indicate this in our results. All data extracted was without adjustments for potential confounding variables. Therefore, the results presented reflect unadjusted data. P-values were extracted where between group statistical analysis was undertaken. Additionally, where HbA1c was reported as a percentage or mmol/mol, a conversion calculation was undertaken to report both measurement units. Additionally, data extracted from quantitative studies (descriptive or inferential statistics) were ‘qualitized’ (i.e., statistical interpretation were transformed into narrative descriptions). These results are presented in a narrative synthesis format [38].

### Methodological quality assessment

All studies were assessed independently by three reviewers (JP, ST, and CKA) for methodological quality using the JBI’s critical appraisal checklists [43]. The critical appraisal checklists included risk of bias for RCTs, quasi-experimental studies, case control, case report, and qualitative research. The checklists included 8 to 13 items with 4 response options: yes, no, unclear, not applicable. Discrepancies in quality assessment were resolved through regular discussion between the reviewers (JP, ST, and CKA). Where consensus could not be reached, a fourth reviewer (MO) decided.

Qualitative data synthesis was conducted using Clarke and Braun’s reflexive thematic analysis method to develop common categories between included studies [44, 45]. Firstly, free line-by-line coding of the primary study’s findings was undertaken. This was followed by the organisation of ‘free codes’ into ‘descriptive’ themes. This process was further underpinned by Dahlgren and Whitehead's determinants of health model which considers a multitude of influences on behaviour related to health [46].

Following the synthesis of both quantitative and qualitative evidence, a convergent integration of these findings was undertaken. Quantitative and qualitative data often address different aspects of a target phenomenon therefore, they may not be capable of confirming or refuting each other, instead their complementarity can be assessed [47]. Moreover, where quantitative and qualitative data appeared related to each other linking observations with explanations, the term ‘complement’ was used as this strengthened the understanding of the phenomenon. Where observations and explanations seemed to oppose each other, the term ‘conflict’ was stated. In the instance, where there were no links, the authors used the term ‘unexplained’ and recommended further research to explain the disparity.

## Results

### Search outcomes

We identified a total of 4,479 records from seven databases. We retrieved 97 of them for full-text screening and 17 studies met the inclusion criteria. Fifteen studies were used for our quantitative analysis [7, 9–22]. The database search for the qualitative studies identified three papers for full-text review (*n* = 2 qualitative studies [48, 49] and *n* = 1 the qualitative component of a mixed methods study [21].

**Fig. 1:**
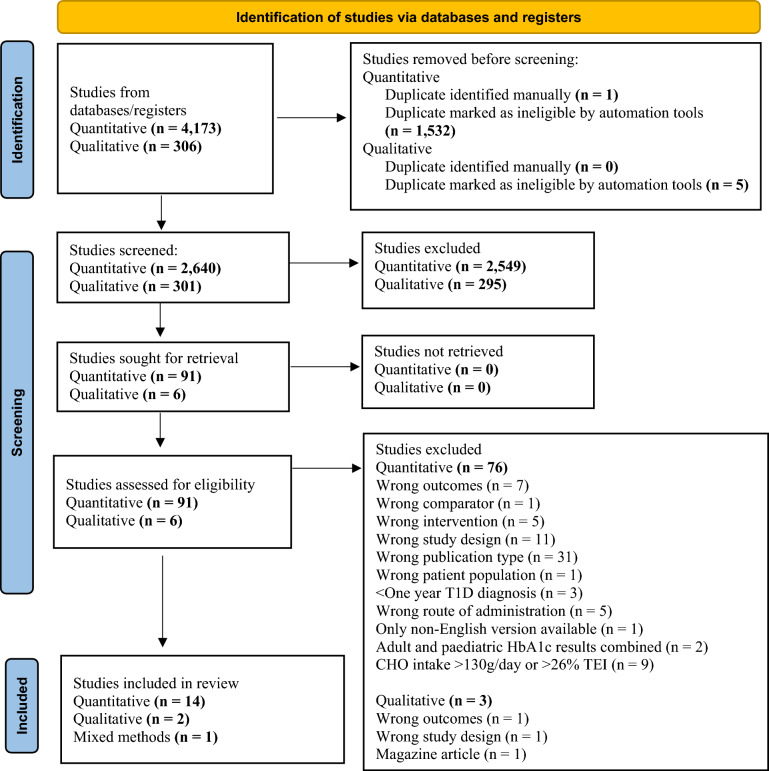
PRISMA flow diagram [37]

Most commonly, studies were excluded due to being the wrong publication type (i.e., thesis, editorals, secondary studies (such as reviews and meta-analysis), conference papers, discussion papers with no original data, and grey literature) and did not meet the definition of a VLCD or LCD, or failed to report both pre and post intervention results relevant to this review (i.e., HbA1c). Inter-rater reliability agreement using Cohen’s kappa (*k*) resulted in almost perfect agreement between reviewers (*k* = 0.81 (95%CI 0.67 to 0.95), *P* < 0.05. The PRISMA flow diagram is shown in Fig. 1.

### Quantitative and qualitative studies characteristics

Included quantitative studies were published from 1992 to 2023 [19, 22] and were conducted in the USA (*n* = 3), [9, 14, 17], UK (*n* = 1), [10] Europe (*n* = 6), [7, 11–13, 16, 20] Australia (*n* = 4), [15, 19, 21, 22], and New Zealand (*n* = 1) [18]. There were three RCTs, [7, 16, 18], three quasi-experimental [12, 19, 20], two case series (studies that grouped together similar case studies/reports) [14, 22], six case reports (studies that included one participant) [9–11, 13, 15, 17], and one mixed methods study [21]. The sample sizes in the quantitative studies varied from one to 48 adults [9–11, 13, 15, 17, 20], with a total of 188 participants included. Participants mean age was 38 years (20–52 years), with a diabetes duration of 21 years (14–41 years), and a baseline mean HbA1c of 8.6% (6.4–16.8%) [70 mmol/mol (46-160 mmol/mol)]. Diet intervention duration ranged from one week to four years and ten months [15, 16]. Dietary intervention characteristics included nine studies which examined a VLCD [9–17] and six LCD [7, 18–22]. The study baseline characteristics of included quantitative studies are shown in Table 1.

**Table 1:** Baseline characteristics of the included quantitative studies by study type

Author, Year, Country	Diet type VLCD or LCD	Study duration	Sample size (*n*)	Male (%)	Age (y)	T1D duration (y)	Body mass index (kg/m^2^)	HbA1c (%)
Randomised control trial								
Krebs et al. 2016, New Zealand [18]	LCD	12wks	5	80	44.5 ± 10.4	23.6 ± 9.9	27.5 ± 2.2	7.9 ± 0.9
Ranjan et al. 2017, Denmark [16]	VLCD	1wk	10	70	48 ± 10	23 ± 7	24.8 ± 1.9	7.0 ± 0.6
Schmidt et al. 2019, Denmark [7]	LCD	12wks	14	43	44 ± 12	19.0 (13.0–22.0)	25.0 (23.7–26.4)	7.3 ± 0.5
Quasi-experimental								
Kleiner et al. 2022, Italy [12]	VLCD	12mths	33	30	41.6 ± 15.0	14.3 ± 11.3	23.9 ± 3.6	8.3 ± 1.7
^b^Ireland, O’Dea, & Nankervis, 1992, Australia [19]	LCD	2wks	8	38	36.0 ± 3.6	23.7 ± 2.6	23.9 ± 1.3	11.1 ± 0.6
Nielsen et al. 2012, Sweden [20]	LCD	4y	48	35	52 ± 11.5	24.0 ± 12.0	25.9 ± 3.5	7.6 ± 1.0
Paul et al. 2022, Australia [21]	LCD	12wks	22	36	43.2 ± 14.7	17.1 ± 13.2	27.2 ± 1.4	8.0 ± 1.7
Case series (retrospective)								
^a^O’Neill et al. 2003, USA [14]	VLCD	18.7 (8.0–61.0)mths	10	50	42.3 ± 13.5	21.1 ± 10.4	NR	6.8 ± 1.1
Turton et al. 2023, Australia [22]	LCD	16wks	26	50	42.8 ± 13.9	21.2 ± 10.4	31.9 ± 5.9	7.7 ± 0.5
Case reports (retrospective)								
Buehler et al. 2021, USA [9]	VLCD	2y	1	100	31	14	30.4	7.7
Eiswirth, Clark, & Diamond, 2018, UK [10]	VLCD	5mths	1	0	20’s	≥ 15	NR	7.5
Gardemann, Knowles, & Marquardt, 2023, Germany [11]	VLCD	9mths	1	100	20’s	≥ 18	19.9	7.2
Kwiendacz et al. 2019, Poland [13]	VLCD	5mths	1	0	28	15	NR	6.4
Raab, 2003, Australia [15]	VLCD	4.8y	1	100	NR	41	NR	8.4
^a^Vernon et al. 2003, USA [17]	VLCD	3mths	1	0	38	NR	20.5	16.8

*g* grams, *HbA1c* glycated haemoglobin*, LCD* low carbohydrate diet (< 130 g/day or < 26% total energy intake), *mth* month, *NR* not reported, *RCT* randomised control trial, *T1D* type 1 diabetes, *T2D* type 2 diabetes, *VLCD* very low carbohydrate diet (≤ 50 g/day or < 10% total energy intake), *wks* weeks, *y* years

^a^Participants with T1D and T2D in this study – only T1D participant results are reported in this review

^b^This study contained two interventions. One intervention used a low fat, low carbohydrate diet and the other used a high fat, low carbohydrate diet. The high fat, low carbohydrate diet intervention did not meet the definition of a low carbohydrate diet (< 130 g/day or < 26% total energy intake) and was excluded from this review. The low fat, low carbohydrate diet intervention did meet the definition of a low carbohydrate diet and was therefore included in this review [19]

Macronutrient distribution (i.e., carbohydrate, protein, and fat) varied across the studies. Dietary support was provided to participants by either nutrition education, meal plans, or food supplies (i.e., nine out of seventeen studies) [7, 14, 16–22] (Additional file 5).

In addition, the included qualitative studies were published from 2015 to 2022 [21, 48, 49]. The three studies (*n* = 2 qualitative studies [48, 49] and *n* = 1 mixed methods [21]) analysed data thematically. The qualitative studies were conducted in Canada (*n* = 1) [48], and New Zealand (*n* = 1) [49]. The mixed methods study was undertaken in Australia [21]. The sample of the qualitative studies included a total of 33 adults ranging from three to 22 participants per study [21, 48, 49]. The demographic data for two of the three studies was incomplete, preventing the aggregate reporting of participant mean age, diabetes duration, and baseline HbA1c [48, 49]. The qualitative studies (*n* = 2) included one that explored the use of a VLCD [48] and one a LCD [49]. The mixed methods study examined a LCD approach [21]. These study characteristics are presented in Table 2.

**Table 2:** Characteristics of the included qualitative studies

Author, Year, Country	Phenomena of interest/research aim	Participant characteristics (sample, gender, age, diabetes duration)	Study design/study setting	Intervention duration/year of data collection	Findings/conclusions
Cresswell et al. 2015, New Zealand [49]	Investigated the effect of carbohydrate counting education and individual choice of carbohydrate counting intake vs carbohydrate counting education and a restricted carbohydrate intake of 75 g of carbohydrate/day in terms of glycaemic control, QoL, and renal function	*n* = 8/8 with T1D Gender not stated Age not stated Diabetes duration not stated	Qualitative Setting not stated	Intervention duration for the LCD was March to May 2013 Data collected was 2013 (after the intervention)	Participants in the restricted carbohydrate diet group reported challenges with insulin management due to experiencing mealtime insulin resistance as they required a significant increase in bolus insulin. This study highlighted that extra health professional and educational support, maybe needed when individuals are transitioning from their usual carbohydrate dietary intake to a LCD
Paul et al. 2022, Australia [21]	Investigated the association between a LCD (< 100 g/day), HbA1c, and QoL in Australian adults living with T1D	*n* = 22/23 with T1D Gender 36% (M), 64% (F) Age 43.2 ± 14.7 years Diabetes duration 17.1 ± 13.2 years	Qualitative component of a mixed methods study Tertiary hospital in South-East, Queensland, Australia	Intervention duration for the LCD was 12 weeks Data collected year was not stated, however data was collected pre and post the intervention	This study demonstrated that a LCD (< 100 g/day) may be a feasible dietary strategy for those living with T1D because it improved glycaemic control without negatively effecting QoL
Wong et al. 2021, Canada [48]	Explored the perspective of patients with diabetes (T1D and T2D) who have followed a KD relating to reasons for starting the diet, motivators, support systems, sources of information, and challenges	*n* = 3/14 with T1D Gender not stated for those with T1D Age not stated for those with T1D Diabetes duration not stated	Qualitative In-person at location convenient for the participant (e.g., home, workplace, or coffee shop) in Montréal, Québec	Intervention duration for the KD was 5(3 to 19) months Data collection occurred during the summer and fall months, 2018	The overall experience of the participants was positive where benefits of the diet included improved glycaemic control, diabetes medication reduction, and weight loss. The benefits appeared to outweigh the challenges (i.e. a lack of support from their healthcare team and informational resources on the KD). This study highlighted that health professional and educational support is needed to help patients make informed decisions, guiding them to safely initiate or discontinue the KD and ensure regular follow up, and monitoring while using a KD

*HbA1c* glycated haemoglobin, *KD* ketogenic diet, *LCD* low carbohydrate diet, *QoL* quality of life, *T1D* type 1 diabetes, *T2D* type 2 diabetes, *vs* versus

### Methodological quality

Using the JBI critical appraisal checklists, Additional File 6 provides a summary of the critical evaluation and assessment of the methodological quality of the included studies. RCTs scores ranged from eight to nine out of ten, quasi-experimental studies scores ranged from six and a half to seven and a half out of ten, case reports scores ranged from six and half to eight out of eight, case series scores ranged from nine to ten out of ten, and qualitative studies score ranged from seven to seven and a half out of ten. These scores reflect how well each study has addressed the possibility of bias in its design, conduct and analysis [43]. The greater the score, the better the methodological quality [43].

### Quantitative studies primary and secondary outcomes

#### Primary outcome: HbA1c

Examination of the primary outcome HbA1c for the quantitative studies showed an average mean difference (improvement) pre- to post-intervention for VLCD and LCD studies that was 2.9% [9–17] and 0.4% [7, 18–22] respectively. According to the American Diabetes Association and the National Institute for Health and Clinical Excellence treatment guidelines, a 0.5% change in HbA1c is considered clinically significant [50, 51]. Hence, a clinically significant impact of VLCDs on HbA1c was evident in eight studies [9–11, 13–17]. One VLCD study reported a statistically significant improvement in HbA1c [12]. Three out of six LCD studies found a statistically significant improvement on HbA1c [20–22]. One LCD study demonstrated a favourable clinical improvement in HbA1c [18] whilst the remaining LCD studies (*n* = 2/6) reported no change in HbA1c [7, 19]. The quantitative studies’ outcomes for VLCDs and LCDs are presented in Tables 3, 4 respectively.

**Table 3:** Very low carbohydrate diet (≤ 50 g/day or < 10% total energy intake) quantitative studies outcomes

Author, year, country	Pre intervention	Post intervention	P-value
Primary outcome			
HbA1c (%)			
Buehler et al. 2021, USA [9]	7.7 ± NR	5.5 ± NR	NR
Eiswirth, Clark & Diamond, 2018, UK [10]	7.5 ± NR	5.3 ± NR	NR
Gardemann, Knowles & Marquardt, 2023, Germany [11]	7.2 ± NR	5.1 ± NR	NR
Kleiner et al. 2022, Italy [12]	8.3 ± 1.7	6.8 ± 0.8	< 0.001
Kwiendacz et al. 2019, Poland [13]	6.4 ± NR	5.4 ± NR	NR
^a^O’Neill et al. 2003, USA [14]	6.8 ± 1.1	5.5 ± 0.8	NR
Raab, 2003, Australia [15]	8.4 ± NR	5.6 ± NR	NR
Ranjan et al. 2017, Denmark [16]	7.0 ± 0.6	6.2 ± 0.4	NR
^a^Vernon et al. 2003, USA [17]	16.8 ± NR	5.3 ± NR	NR
Secondary outcomes			
Bolus insulin (units/day)			
Buehler et al. 2021, USA [9]	33 ± NR	1 ± NR	NR
Eiswirth, Clark & Diamond, 2018, UK [10]	NR	NR	NR
Gardemann, Knowles & Marquardt, 2023, Germany [11]	20–24 ± NR	12.5 ± NR	NR
Kleiner et al. 2022, Italy [12]	18.3 ± 9.5	10.3 ± 6.5	< 0.001
Kwiendacz et al. 2019, Poland [13]	NR	14.4 ± NR	NR
^a^O’Neill et al. 2003, USA [14]	NR	NR	NR
Raab, 2003, Australia [15]	NR	NR	NR
Ranjan et al. 2017, Denmark [16]	16.3 ± 7.9	6.6 ± 1.8	0.001
^a^Vernon et al. 2003, USA [17]	NR	NR	NR
Weight (kg)			
Buehler et al. 2021, USA [9]	NR	NR	NR
Eiswirth, Clark & Diamond, 2018, UK [10]	NR	Decreased by 3	NR
Gardemann, Knowles & Marquardt, 2023, Germany [11]	61 ± NR	61 ± NR	NR
Kleiner et al. 2022, Italy [12]	68.9 ± 13.5	66.0 ± 6.8	NS
Kwiendacz et al. 2019, Poland [13]	NR	62 ± NR	NR
^a^O’Neill et al. 2003, USA [14]	NR	NR	NR
Raab, 2003, Australia [15]	84.0 ± NR	72 ± NR	NR
Ranjan et al. 2017, Denmark [16]	75.2 ± 11.7	72.9 ± 10.3	NR
^a^Vernon et al. 2003, USA [17]	61.2 ± NR	69.4 ± NR	NR
Quality of life (participant reported)			
Buehler et al. 2021, USA [9]	NR	NR	NR
Eiswirth, Clark & Diamond, 2018, UK [10]	NR	Improved	NR
Gardemann, Knowles & Marquardt, 2023, Germany [11]	NR	Improved	NR
Kleiner et al. 2022, Italy [12]	NR	NR	NR
Kwiendacz et al. 2019, Poland [13]	NR	NR	NR
^a^O’Neill et al. 2003, USA [14]	NR	NR	NR
Raab, 2003, Australia [15]	NR	Improved	NR
Ranjan et al. 2017, Denmark [16]	NR	NR	NR
^a^Vernon et al. 2003, USA [17]	NR	NR	NR

*HbA1c glycated haemoglobin, kg kilograms, NR not reported, NS not significant, T1D type 1 diabetes, T2D type 2 diabetes*

^*a*^*Participants with T1D and T2D in this study – only T1D participant results are reported in this review*

**Table 4:** Low carbohydrate diet (< 130 g/day or < 26% total energy intake) quantitative studies outcomes

Author, year, country	Pre intervention	Post intervention	P-value
Primary outcome			
HbA1c (%)			
Krebs et al. 2016, New Zealand [18]	7.9 ± 0.9	7.2 ± 0.4	NS
^a^Ireland, O’Dea & Nankervis, 1992, Australia [19]	11.1 ± 0.6	11.6 ± 0.8	NS
Nielsen et al. 2012, Sweden [20]	7.6 ± 1.0	6.9 ± 1.0	< 0.001
Paul et al. 2022, Australia [21]	8.0 ± 1.7	7.1 ± 1.1	0.003
Schmidt et al. 2019, Denmark [7]	7.3 ± 0.5	7.4 ± 0.4	NS
Turton et al. 2023, Australia [22]	7.7 ± 0.5	7.1 ± 0.7	< 0.01
Secondary outcomes			
Bolus insulin (units/day)			
Krebs et al. 2016, New Zealand [18]	NR	NR	NR
^a^Ireland, O’Dea & Nankervis, 1992, Australia [19]	NR	NR	NR
Nielsen et al. 2012, Sweden [20]	NR	NR	NR
Paul et al. 2022, Australia [21]	20.3 ± 6.7	13.4 ± 6.5	< 0.0001
Schmidt et al. 2019, Denmark [7]	NR	15.1 ± 4.4	NR
Turton et al. 2023, Australia [22]	NR	NR	NR
Weight (kg)			
Krebs et al. 2016, New Zealand [18]	83.2 ± 11.0	78.0 ± 6.4	NS
^a^Ireland, O’Dea & Nankervis, 1992, Australia [19]	62.1 ± 3.1	61.9 ± 3.1	NS
Nielsen et al. 2012, Sweden [20]	77.6 ± 15	76.7 ± 14.6	NS
Paul et al. 2022, Australia [21]	79.3 ± 11.1	77.4 ± 11.3	0.013
Schmidt et al. 2019, Denmark [7]	77.4 ± 10.6	75.5 ± 10.9	0.012
Turton et al. 2023, Australia [22]	93.8 ± 18.7	91.4 ± 17.7	< 0.025
Quality of life (participant reported)			
Krebs et al. 2016, New Zealand [18]	NR	NR	NR
^a^Ireland, O’Dea & Nankervis, 1992, Australia [19]	NR	NR	NR
Nielsen et al. 2012, Sweden [20]	NR	NR	NR
Paul et al. 2022, Australia [21]	41.6 ± 11.2	40.5 ± 14.3	NS
Schmidt et al. 2019, Denmark [7]	30.9 ± 3.8	27.1 ± 6.5	NS
Turton et al. 2023, Australia [22]	33.8 ± 5.8	30.3 ± 7.4	< 0.025

*HbA1c* glycated haemoglobin*, kg* kilograms, *NR* not reported, *NS* not significant

^a^This study contained two interventions. One intervention used a low fat, low carbohydrate diet and the other used a high fat, low carbohydrate diet. The high fat, low carbohydrate diet intervention did not meet the definition of a low carbohydrate diet (< 130 g/day or < 26% total energy intake) and was excluded from this review. The low fat, low carbohydrate diet intervention did meet the definition of a low carbohydrate diet and was therefore included in this review [19]

#### Secondary outcome: bolus insulin

The overall influence of a VLCD and LCD on bolus insulin was largely missing from the published studies, making it difficult to draw conclusive outcomes. Very low carbohydrate diet studies (*n* = 4/9) reported a reduction in bolus insulin [9, 11, 12, 16], one VLCD study reported only post intervention bolus insulin dose [13], and *n* = 4/9 studies did not report bolus insulin [10, 14, 15, 17]. Low carbohydrate diet studies (*n* = 1/6) also showed a reduction in bolus insulin requirements [21]. Only one LCD study (*n* = 1/6) reported post intervention bolus insulin [7] and *n* = 4/6 LCD studies did not report this outcome [18–20, 22].

#### Secondary outcome: weight

Overall, weight change varied with VLCDs and LCDs. Three of nine VLCD studies reported participant weight reduction (pre to post intervention) ranging from 3 to 14% respectively [12, 15, 16]. The results of one VLCD study showed a 13% weight gain (*n* = 1 participant) [17], while another VLCD study demonstrated no weight change pre to post intervention [11]. Two VLCD studies did not include this outcome [9, 14], and two studies only provided weight measurements post intervention [10, 13]. Studies that examined a LCD (*n* = 5/6) demonstrated weight reduction (pre to post intervention) ranging from 1 to 6% [7, 18, 20–22]. One LCD study (*n* = 1/6) reported no weight change [19].

#### Secondary outcome: quality of life

Furthermore, the influence of a VLCD (*n* = 3/9 studies) [10, 11, 15] and LCD (*n* = 3/6 studies) [7, 21, 22] on QoL showed a favorable effect. Six of the nine VLCD studies [9, 12–14, 16, 17] and *n* = 3/6 LCD studies did not report or assess this outcome [18–20].

### Qualitative outcomes

Examination of qualitative studies data explored the perceptions, knowledge, and experience of adults living with T1D. Several themes were identified and reported under the categories of either enablers or barriers to the adoption of a VLCD or LCD. The enablers included themes relating to (1) motivation to follow the diet and (2) health benefits of the diet. The barriers comprised of (3) challenges to the diet and (4) limited information (i.e., participants knowledge, information sources) about the diet. Direct quotes were extracted to illustrate a range of different participant views and experiences which act as evidence to support the below commentary and this study’s findings.

### Enablers—for individuals with T1D adhering to a VLCD or LCD

#### Motivation to follow the diet

Motivation was key to several participants engaging with and adhering to VLCD or LCD eating regimens across all three studies [21, 48, 49]. For many these diets were not difficult to follow as one participant claimed: *“because you know…. a steak with a salad, it’s super good and ….it’s fine”* [48].

A few participants were impressed with the availability of specialty food products such as: *“low-carb bread”* which motivated them to keep going with the diet [49].

Because of the diet one participant felt empowered to: *“say no to a lot of sweet things.… (due to not having cravings) anymore which she further added “helps me mentally”* [21].

While another participant felt that once they were equipped with the skills to follow a LCD properly this led: “*to a sense of awareness…. (which was) quite empowering”* [49].

Others were motivated to continue with the diet beyond the study period because it simply made them feel: “*fantastic”* [49] in addition to providing a *“drastic…. (but positive) difference” to their lives* [48].

#### Health benefits of the diet

Overall, many participants across all three studies reported feeling healthier while following a VLCD or LCD as one participant stated: “*I just feel better and all my family and my partner have said I just seem better”* [21].

For some participants following a VLCD or LCD led to weight loss *“which was an unexpected benefit”* [49] and made them feel *“good”* [21].

Several other advantages were highlighted with one participant exclaiming: *“it made me sleep better, I was less tired, and more focussed”* [21].

The diet was also recognised to assist with: *“keeping blood glucose levels in range……. and for longevity of life”* [21].

Need for insulin went down for some with the following participant describing the health advantage as: *“Normally, at meals I would take between 14 and 16 units of insulin……. With the ketogenic diet….. I usually give myself between 4 and 6 units. So, it’s 4 times less”* [48].

Likewise, another participant stated: *“I won’t be going back to the pump in a hurry (as now I need less insulin) and will continue on the low carb diet”* [21].

Part of feeling “normal” is the ability to “fit in” especially regarding social occasions. For example, in relation to eating out it was acknowledged that: “*there is always something on the menu which is accessible (for a ketogenic diet)”* [48].

### Barriers—for individuals with T1D adhering to a VLCD or LCD

#### Challenges of the diet

Adjusting meal time insulin to carbohydrate ratio proved to be an initial challenge for many across the three studies due to unexpected variation in glycaemia i.e., going too low due to too much insulin or going to high due to too little insulin. Participants reported needing to adjusted their insulin to carbohydrate ratio frequently at the beginning of the intervention due to the reduced carbohydrate intake to achieve optimal glycaemia as their usual ratio was inappropriate [21, 48, 49]. The following participant reported:

*“my ratio’s had to change quite quickly”* due to the reduction in dietary carbohydrate being consumed [49].

It was reported that reading information on food products can be confusing and burdensome for participants [49]. In addition, one participant made the point that: *“the low carb diet can be restrictive”* [21], highlighting the emotional burden patients with T1D face in daily living with the disease.

It was also acknowledged by some participants that the diet negatively impacted their mood when it changed from an unrestricted diet to a LCD and applying carbohydrate counting skills: *“food from a pleasure to chemistry … suddenly enjoyment of meals went suddenly down”* [49].

Due to the nature of low carbohydrate eating, it can be a difficult regimen to maintain [21, 48]. From a social perspective this point was emphasised in the following reflection: *“because our society is so high carb, you really need to be mindful…… it (can be) quite restrictive when eating out with friends”* [21].

Having diabetes and managing the condition was described by one participant as like having: *“another child… it was just something I looked after and I didn’t put it in everybody’s faces…. but (this changed when) everybody started seeing me reading labels profusely and measuring things”* [49].

#### Limited information (participants knowledge, information sources) about the diet

While many participants acknowledged the value of following a LCD and carbohydrate counting there were some key concerns relating to insulin to carbohydrate ratio application when following such a diet. One participant stated that despite undertaking carbohydrate training felt there were still skills that needed to be developed stating *“a lot of questions about carbohydrate counting and how it works because there’s quite a few anomalies”* [49].

Participants reported a paucity of authoritative resources for individuals living with T1D who choose to follow a low carbohydrate eating regimen [48]. One participant acknowledged that this prompted them to access a: “*Facebook group called Type 1 Diabetes Quebec…. On there some people posted an article that came from another website—‘Long Live Bacon’”* [48].

### Convergent integrated synthesis findings

A convergent integrated approach was undertaken to identify patterns across all the studies as well as explore relationships of the data between and within the studies [38]. Four themes were developed and included (1) motivation to follow the diet, (2) health benefits of the diet, (3) challenges to the diet and (4) limited information (i.e., participants knowledge, information sources) about the diet that where either enablers or barriers to following a VLCD or LCD. They were further synthesised based on whether a particular aspect of the developed theme appeared to *contradict* or *complement* the initial findings from qualitative and quantitative data.

The key findings relating to each theme are as follows: (1) Participants reported being motivated to follow a VLCD or LCD however this state of motivation did not appear to have a beneficial impact on quantitative outcomes examined (i.e., HbA1c, bolus insulin, weight, and QoL, (2) Participants reported experiencing health benefits of the diets however quantitative findings were contradictory to this fact i.e., showing no improvement, (3) Due to limited research investigating information available to participants who adopt a VLCD or LCD no inter-relational connection could be identified between qualitative and quantitative data, and (4) Participants reported that it was a challenge to follow a VLCD or LCD. This was observed in the quantitative results with no significant improvements in HbA1c, bolus insulin, weight, and QoL reported. Table 5 shows the integrated findings providing insight into the complex inter-relational connections (complementary, conflicting, or unexplained links) between quantitative and qualitative results [38, 52].

**Table 5:** Convergent integrated synthesis findings of included quantitative and qualitative studies

Quantitative synthesis (predictor)^a^ vs Quantitative result	Qualitative synthesis (category: enabler or barrier)^b^	Integration (complement^c^ /conflict^d^/unexplained^e^)
HbA1c (%) Quantitative result: ^f^Four out of fifteen studies show that VLCDs [12] and LCDs [20–22] contributed to improved HbA1c levels in adults living with T1D. Nonetheless, we cannot conclude that these diets result in optimal management of HbA1c	Enabler Theme: Motivation to follow the diet Adults with T1D appear to be motivated to follow a VLCD or LCD as it improved HbA1c	The predictor of effective glycaemic control was conflicted by the qualitative data which found adults with T1D are motivated to follow a VLCD or LCD to improve their HbA1c
	Enabler Theme: Health benefits of the diet Adults with T1D experienced health benefits such as improved sleep and mood when using a VLCD or LCD	The predictor of effective glycaemic control was conflicted by the qualitative data which found adults with T1D believe following a VLCD or LCD improves health outcomes
	Barrier Theme: Challenges to the diet Adults with T1D found the effect of BGL variation difficult to manage when following a VLCD or LCD	The predictor of effective glycaemic control was complemented by the qualitative data which reported adults with T1D found fluctuations in blood glucose was difficult to manage with a VLCD or LCD
	Barrier Theme: Limited information (participants knowledge, information sources) about the diet NULL	The predictor of effective glycaemic control was unexplained because there was no qualitative data exploring availability of information on a VLCD or LCD to improve HbA1c in adults living with T1D
Bolus insulin (units/day) Quantitative result: Quantitative result: ^f^Three out of fifteen studies show that in adults living with T1D, VLCDs [12, 16] and LCDs [21] did result in reducing bolus insulin (units/day). Nonetheless, we cannot conclude that these diets reduce bolus insulin (units/day)	Enabler—Motivation to follow the diet NULL	The predictor of bolus insulin was unexplained because there was no qualitative data exploring motivation to follow a VLCD or LCD to reduce insulin needs in adults living with T1D
	Enabler—Health benefits of following the diet Adults with T1D recognise the need for reduction to bolus insulin when following a VLCD or LCD	The predictor of bolus insulin administration was conflicted by the qualitative data which found adults with T1D feel that a beneficial outcome is the need for less insulin when following a VLCD or LCD
	Barrier—Challenges with the diet Adults with T1D found it challenging to manage bolus insulin when following a VLCD or LCD	The predictor of bolus insulin administration was complemented by the qualitative data which found adults with T1D find it challenging to manage their bolus insulin when following a VLCD or LCD
	Barrier—Limited information (participants knowledge, information sources) about the diet Adults with T1D had limited information in relation to managing bolus insulin when following a VLCD or LCD	The predictor of bolus insulin administration was complemented by the qualitative data which found adults with T1D have limited access to information about managing bolus insulin when following a VLCD or LCD
Weight (kg) Quantitative result: ^f^Three out of fifteen studies show that in adults living with T1D, a LCD did achieve weight loss [7, 21, 22]. Nonetheless, we cannot conclude that LCDs result in weight loss No statistical analysis was undertaken for VLCDs and weight	Enabler Theme: Motivation to follow the diet Adults with T1D were motivated to follow a VLCD or LCD as it facilitated desired weight loss	The predictor of weight was conflicted by the qualitative data which found adults with T1D are motivated to follow a VLCD or LCD to facilitate (desired) weight loss
	Enabler Theme: Health benefits of the diet Adults with T1D recognised the health benefit of weight loss when following a VLCD or LCD	The predictor of weight was conflicted by the qualitative data which found adults with T1D recognise that following a VLCD or LCD can facilitate (desired) weight loss
	Barrier Theme: Challenges to the diet NULL	The predictor of weight was unexplained because there was no qualitative data exploring challenges of following a VLCD or LCD to facilitate (desired) weight loss in adults living with T1D
	Barrier Theme: Limited information (participants knowledge, information sources) about the diet NULL	The predictor of weight was unexplained because there was no qualitative data exploring availability of information on a VLCD or LCD to facilitate (desired) weight loss in adults living with T1D
Quality of Life (QoL) Quantitative result: ^f^One out of fifteen studies show that in adults living with T1D, a LCDs improved QoL [22]. Nonetheless, we cannot conclude that a LCD improves or reduces QoL No statistical analysis was undertaken for VLCDs and Q oL	Enabler—Motivation to follow the diet Adults with T1D were motivated to follow a VLCD or LCD as it made them feel better	The predictor of QoL was conflicted by the qualitative data which found adults with T1D are motivated to follow a VLCD or LCD as it made them feel better
	Enabler—Health benefits to following the diet Adults with T1D experienced positive health benefits such as better sleep and mood when following a VLCD or LCD	The predictor of QoL was conflicted by the qualitative data which found adults with T1D experienced health benefits while following a VLCD or LCD
	Barrier—Challenges to the diet Adults with T1D felt that a VLCD or LCD can be restrictive to their lifestyle	The predictor of QoL was complemented by the qualitative data which found adults with T1D found it difficult to follow a VLCD or LCD
	Barrier—Limited information (participants knowledge, information sources) about the diet NULL	The predictor of QoL was unexplained because there was no qualitative data exploring the availability of information in relation to following a VLCD or LCD

*BGL* blood glucose level, *HbA1c* glycated haemoglobin, *kg* kilograms, *LCD* low carbohydrate diet, *QoL* quality of life*, T1D* type 1 diabetes, *VLCD* very low carbohydrate diet

^a^Predictor in the context of the integrated synthesis of quantitative and qualitative study findings, the predictors include HbA1c, bolus insulin, weight, and QoL

^b^Enabler, barrier are the overarching categories that influenced adherence to a very low or low carbohydrate diet

^c^Complement is related to links between observation and explanation of quantitative and qualitative findings

^d^Conflict is related to opposing links between observation and explanation of quantitative and qualitative findings

^e^Unexplained is related to no links between observation and explanation of quantitative and qualitative findings

^f^Findings from statistical analysis

## Discussion

This Mixed Methods Systematic Review (MMSR) has addressed an important gap in the research relating to firstly the effectiveness of a VLCD and LCD on HbA1c in adults living with T1D; and secondly the enablers and barriers to following a VLCD or LCD amongst adults living with T1D. In addition, it shows that a decrease in HbA1c of ≥ 0.5% is associated with other health benefits such as a reduction in bolus insulin [9, 11, 12, 16, 21], decrease in weight [10, 12, 15, 16, 18, 20–22], and an improved QoL [10, 11, 15, 21, 22]. Hence, these diets may be considered a useful strategy in the management of HbA1c for adults living with T1D. In addition, our qualitative findings addressed the gap in evidence related to the lived experiences of those with T1D who have chosen to adopt a VLCD or LCD. We found that individuals were motivated to follow a VLCD or LCD, because they believed these dietary regimens would improve their HbA1c whilst attaining weight reduction [21, 48, 49] increased vitality [21, 48, 49], and a better QoL [21, 48, 49]. Furthermore, the challenges that emerged with adopting these diets included difficulties with managing BGL variations, limited information on how to handle bolus insulin in response to the dietary changes, and a feeling of having limited foods choices when eating out [21, 48, 49]. Nonetheless, the overall quantitative and qualitative findings do not support each other due to a lack of conclusive evidence on this topic. Further mixed methods studies are needed to provide more definitive evidence.

The influence of VLCD or LCD on bolus insulin, the first of this reviews’ three secondary outcomes, was not commonly reported despite its connection to carbohydrate intake. This result was anticipated due to the fact that mealtime insulin is primarily influenced by the quantity of carbohydrates consumed [12, 16]. Previous research conducted by Stamati et al. [24] reported that bolus insulin decreased (mean difference = − 8.61 units/day, 95% CI (− 16.39 to − 0.82, *I*^2^ = 80%, *P* =  < 0.01) in participants with T1D who used a LCD. According to research, a reduction in bolus insulin has been associated with weight loss among adults living with T1D [53]. This suggests that weight loss could be a potential benefit of adopting a VLCD or LCD, as observed in both our review and the literature [12, 16, 21].

Moreover, VLCD and LCD studies found participant weight reduction ranged from 3 to 14% [12, 15, 16] and 1 to 6% [7, 20–22] respectively. Weight reduction may be related to several factors such as reduced carbohydrate intake [7, 10, 12, 15, 16, 18, 20–22], decreased hunger, and increased satiety due to changes in overall protein and/or fat which generally occur with a decrease in carbohydrate intake [7]. In addition, physical activity [16] and/or reduced insulin needs can influence weight loss [21, 24, 54]. Many participants reported experiencing health benefits when following a VLCD or LCD such as improved glycaemia, desired weight loss, an increased perceived energy levels, and a more optimistic outlook on life [15, 21, 48, 49]. Therefore, the interpretation of this finding could also be that weight loss is associated with the improved QoL and the use of a VLCD or LCD [7, 15, 21, 22].

Participants in this review lamented having limited access to relevant information. In many cases, health professionals were either unwilling or unable to provide support regarding initiation and maintenance of VLCDs or LCDs resulting in social media being suggested as a viable alternative [48]. However, the credibility of such media may be an issue [48]. Such concerns have been reported previously [55]. Nevertheless, if individuals experience benefit following a VLCD or LCD, no matter what the underlying mechanism for success is, they are more likely to continue. Consequently, the availability of credible information is imperative to ensure that optimal health outcomes are achieved in both the short and long term.

Quality of life (QoL) was not consistently reported in all studies. However, the overall trend showed that both VLCDs and LCDs did not have a detrimental effect on participants' QoL [7, 10, 11, 15, 21, 22]. Despite this finding, T1D has been linked with reduced QoL due to the constant demands of self-management [33, 56]. This review found a VLCD or LCD can be socially isolating, restrictive due to fewer food choices at restaurants, difficulties with participating in celebrations, while on holidays, or travelling, and change in mealtime insulin needs [21, 48, 49, 57]. Such factors may also contribute to reducing QoL by impacting social normalcy. In contrast, some participants in this review reported that they found following a VLCD or LCD easy while others experienced the opposite [21, 48]. Hence, an individualised, patient-centred approach may be beneficial to overcome these challenges. The findings of this review reveal inconclusive and contradictory evidence relating to QoL and the use of a VLCD or LCD, emphasizing the requirement for further research.

It could be argued that as participants chose to partake in a VLCD or LCD intervention almost all had some form of motivation to commence aiding their ability to adhere to the diet. Nevertheless, this motivation manifested for participants in different ways including the belief that the diet was easy to follow in addition to being satisfying and enjoyable plus something they could control which was personally empowering [49]. Ultimately, for most participants they were motivated to follow the diet as it made them feel mentally and/or physically better [21, 48, 49]. A recent scoping review by Sarfo et al. [58] examined the self-determination theory (SDT) in relation to key management strategies for adults with all types of diabetes. The authors reported that SDT improved QoL, diabetes treatment adherence, and self-management [58]. Likewise, the authors used Dahlgren and Whitehead's determinants of health model to theoretically underpin this review as it acknowledges the wide range of factors that influence health-related behaviour [46]. Where patients with T1D are motivated to try a VLCD or LCD health professionals can utilise this opportunity to inform them about the diets and support their decision by giving medical nutrition therapy guidance and strategies to manage potential barriers that may be encountered to assist achieving positive diabetes and health outcomes [48, 59, 60].

### Strengths and limitations

The strengths of this review are that as far as the authors are aware, this is the first Mixed Methods Systematic Review to be published regarding the topic. This novel approach comprehensively synthesizes the current evidence beyond what a single review method can offer by combining findings on the effectiveness (quantitative evidence) and perceptions, knowledge, and experience (qualitative evidence) of adults living with T1D who have adopted a VLCD or LCD. In addition, conducting a convergent integrated synthesis of data of this topic led to a deeper understanding of this research area which is a major strength of this review [61].

Nevertheless, some limitations associated with this review should be acknowledged. The decision to only include peer-reviewed journal articles may have led to inadvertent exclusion of grey literature in this important area of clinical practice. However, the exploration of seven prominent health databases provided a comprehensive overview of the topic. The lack of blinding in dietary interventions, and the fact that highly motivated people living with T1D often volunteer to participate in studies may have influenced some outcomes [20]. The limited number of included studies, small sample sizes, varied methodological quality, inconsistency in outcomes reported, and varied study durations in this review highlights the substantial heterogeneity between the included studies and the gaps in care for individuals living with T1D who choose to follow a VLCD or LCD. These limitations indicate the current paucity of knowledge in addition to stressing the need for more rigorous investigations which examine both the effectiveness and experiences of these diets in those living with T1D when used over a long time.

### Future directions—implications for research and clinical practice

To-date, there is a dearth of research on this topic. In this review, the included quantitative studies yielded inconclusive results for both primary and secondary outcomes (HbA1c, bolus insulin, weight, and QoL). In addition, the small number of qualitative studies included reflects a paucity of research related to the experiences of individuals living with T1D while following a VLCD or LCD. Future research such as RCTS and mixed methods studies involving larger participant samples over longer durations are needed to provide more definitive evidence in relation to the efficacy of VLCDs or LCDs. In addition, more high-quality studies can better inform clinical practice given that healthcare is such a complex phenomenon, understanding associations and outcomes in addition to experiences and perceptions. Further evidence could enable diabetes health professionals to better support patients who choose to adopt a VLCD or LCD to achieve optimal health and wellbeing.

## Conclusions

This mixed methods systematic review aimed to investigate the effectiveness of a VLCD and LCD on HbA1c while exploring the perceptions, knowledge, and experiences of adults living with T1D who have followed a VLCD or LCD. This review encompasses all relevant quantitative and qualitative findings on VLCDs and LCDs for adults living with T1D. This review lacks sufficient evidence to definitively ascertain the effectiveness of VLCDs and LCDs in achieving significant improvements in HbA1c for adults with T1D, contradictory to the personal experiences of many participants.

## Supplementary Information

Additional file 1: PRISMA 2020 checklist.

Additional file 2: MEDLINE (Ovid) search strategy for included studies (1946 to 1 October 2023).

Additional file 3: Inclusion and exclusion criteria for selection of quantitative and qualitative studies.

Additional file 4: Key variables extracted from included quantitative and qualitative studies.

Additional file 5: Intervention calories, macronutrient distribution, and dietary support methods of included quantitative studies.

Additional file 6: Quality appraisal of studies.

## Data Availability

All data generated or analysed during this study are included in this published article and its additional information files.

## Abbreviations

BGL: Blood glucose level
CI: Confidence interval
HbA1c: Glycated haemoglobin
JBI: Joanna Briggs Institute
LCD: Low carbohydrate diet
mmol/mol: Millimoles per mole
PRISMA: Preferred reporting items for systematic reviews and meta-analyses
PROSPERO: International prospective register of systematic reviews
QoL: Quality of life
RCT: Randomised control trial
SDT: Self-determination theory
TEI: Total energy intake
T1D: Type 1 diabetes
T2D: Type 2 diabetes
VLCD: Very low carbohydrate diet

## Declarations

**Ethics approval and consent to participate**

Not applicable.
