# Correction: Low carbohydrate diets, glycaemic control, enablers, and barriers in the management of type 1 diabetes: a mixed methods systematic review

**DOI:** 10.1186/s13098-025-01654-3

**Published:** 2025-03-19

**Authors:** Janine Paul, Rati Jani, Sarah Thorning, Mila Obucina, Peter Davoren, Catherine Knight-Agarwal

**Affiliations:** 1 School of Clinical Science, Faculty of Health, University of Canberra, Bruce, ACT 2617 Australia; 2 Diabetes and Endocrinology, Adult Outpatient Department, Gold Coast University Hospital and Health Service, 1 Hospital Boulevard, D Block, Area 4, Southport, QLD 4215 Australia; 3 School of Health Sciences and Social Work, Griffith University, Southport, QLD 4215 Australia; 4 Education and Research Unit, Central Queensland Hospital and Health Service, Rockhampton, QLD 4700 Australia; 5 Emergency Department, Gold Coast University Hospital and Health Service, Southport, QLD 4215 Australia; 6 School of Medicine and Dentistry, Griffith University, Southport, QLD 4215 Australia

**Correction:** **Diabetology & Metabolic Syndrome (2024) 16:261.** 10.1186/s13098-024-01496-5

In the sentence beginning “Included quantitative studies were published from 1992,…” in this article [1], the text “There were three RCTs, [7, 16, 18], three quasi-experimental [12, 19, 20], three case series (studies that grouped together similar case studies/reports) [14, 17, 22], five case reports (studies that included one participant) [9–11, 13, 15], and one mixed methods study [21]” should have read “There were three RCTs, [7, 16, 18], three quasi-experimental [12, 19, 20], two case series (studies that grouped together similar case studies/reports) [14, 22], six case reports (studies that included one participant) [9–11, 13, 15, 17], and one mixed methods study [21]”.

In the sentence beginning “Examination of the primary outcome ……” in this article, the text “pre- to post-intervention for VLCD and LCD studies that was 2.9% [11–17, 19, 20] and 0.4% [9, 10, 18, 21] respectively” should have read “pre- to post-intervention for VLCD and LCD studies that was 2.9% [9–17] and 0.4% [7, 18–22] respectively”.

In the sentence beginning “Examination of the primary outcome ……” in this article, the text “Hence, a clinically significant impact of VLCDs on HbA1c was evident in eight studies [11–17, 20]” should have read “Hence, a clinically significant impact of VLCDs on HbA1c was evident in eight studies [9–11, 13–17]”.

The Table 3 caption was inadvertently truncated as “Very low carbohydrate diet (≤ 50 g/day or < 10% total energy intake) quantitative” but should read as “Very low carbohydrate diet (≤ 50 g/day or < 10% total energy intake) quantitative studies outcomes quality of life section the reference for Gardemann, Knowles & Marquardt, 2023, Germany reported as number [20]. Corrected to [11]”.

Incorrect Table 3 caption

**Table:** Very low carbohydrate diet (≤ 50 g/day or < 10% total energy intake) quantitative studies outcomes

Author, year, country	Pre intervention	Post intervention	P-value
Primary outcome			
HbA1c (%)			
Buehler et al. 2021, USA [9]	7.7 ± NR	5.5 ± NR	NR
Eiswirth, Clark & Diamond, 2018, UK [10]	7.5 ± NR	5.3 ± NR	NR
Gardemann, Knowles & Marquardt, 2023, Germany [11]	7.2 ± NR	5.1 ± NR	NR
Kleiner et al. 2022, Italy [12]	8.3 ± 1.7	6.8 ± 0.8	< 0.001
Kwiendacz et al. 2019, Poland [13]	6.4 ± NR	5.4 ± NR	NR
^a^O’Neill et al. 2003, USA [14]	6.8 ± 1.1	5.5 ± 0.8	NR
Raab, 2003, Australia [15]	8.4 ± NR	5.6 ± NR	NR
Ranjan et al. 2017, Denmark [16]	7.0 ± 0.6	6.2 ± 0.4	NR
^a^Vernon et al. 2003, USA [17]	16.8 ± NR	5.3 ± NR	NR
Secondary outcomes			
Bolus insulin (units/day)			
Buehler et al. 2021, USA [9]	33 ± NR	1 ± NR	NR
Eiswirth, Clark & Diamond, 2018, UK [10]	NR	NR	NR
Gardemann, Knowles & Marquardt, 2023, Germany [11]	20–24 ± NR	12.5 ± NR	NR
Kleiner et al. 2022, Italy [12]	18.3 ± 9.5	10.3 ± 6.5	< 0.001
Kwiendacz et al. 2019, Poland [13]	NR	14.4 ± NR	NR
^a^O’Neill et al. 2003, USA [14]	NR	NR	NR
Raab, 2003, Australia [15]	NR	NR	NR
Ranjan et al. 2017, Denmark [16]	16.3 ± 7.9	6.6 ± 1.8	0.001
^a^Vernon et al. 2003, USA [17]	NR	NR	NR
Weight (kg)			
Buehler et al. 2021, USA [9]	NR	NR	NR
Eiswirth, Clark & Diamond, 2018, UK [10]	NR	Decreased by 3	NR
Gardemann, Knowles & Marquardt, 2023, Germany [11]	61 ± NR	61 ± NR	NR
Kleiner et al. 2022, Italy [12]	68.9 ± 13.5	66.0 ± 6.8	NS
Kwiendacz et al. 2019, Poland [13]	NR	62 ± NR	NR
^a^O’Neill et al. 2003, USA [14]	NR	NR	NR
Raab, 2003, Australia [15]	84.0 ± NR	72 ± NR	NR
Ranjan et al. 2017, Denmark [16]	75.2 ± 11.7	72.9 ± 10.3	NR
^a^Vernon et al. 2003, USA [17]	61.2 ± NR	69.4 ± NR	NR
Quality of life (participant reported)			
Buehler et al. 2021, USA [9]	NR	NR	NR
Eiswirth, Clark & Diamond, 2018, UK [10]	NR	Improved	NR
Gardemann, Knowles & Marquardt, 2023, Germany [11]	NR	Improved	NR
Kleiner et al. 2022, Italy [12]	NR	NR	NR
Kwiendacz et al. 2019, Poland [13]	NR	NR	NR
^a^O’Neill et al. 2003, USA [14]	NR	NR	NR
Raab, 2003, Australia [15]	NR	Improved	NR
Ranjan et al. 2017, Denmark [16]	NR	NR	NR
^a^Vernon et al. 2003, USA [17]	NR	NR	NR

*HbA1c* glycated haemoglobin, *kg* kilograms, *NR* not reported, *NS* not significant, *T1D* type 1 diabetes, *T2D* type 2 diabetes

^a^Participants with T1D and T2D in this study – only T1D participant results are reported in this review

Correct Table 3 caption

**Table 3:** Very low carbohydrate diet (≤ 50 g/day or < 10% total energy intake) quantitative studies outcomes quality of life section the reference for Gardemann, Knowles & Marquardt, 2023, Germany reported as number [20]. Corrected to [11]

Author, year, country	Pre intervention	Post intervention	P-value
Primary outcome			
HbA1c (%)			
Buehler et al. 2021, USA [9]	7.7 ± NR	5.5 ± NR	NR
Eiswirth, Clark & Diamond, 2018, UK [10]	7.5 ± NR	5.3 ± NR	NR
Gardemann, Knowles & Marquardt, 2023, Germany [11]	7.2 ± NR	5.1 ± NR	NR
Kleiner et al. 2022, Italy [12]	8.3 ± 1.7	6.8 ± 0.8	< 0.001
Kwiendacz et al. 2019, Poland [13]	6.4 ± NR	5.4 ± NR	NR
^a^O’Neill et al. 2003, USA [14]	6.8 ± 1.1	5.5 ± 0.8	NR
Raab, 2003, Australia [15]	8.4 ± NR	5.6 ± NR	NR
Ranjan et al. 2017, Denmark [16]	7.0 ± 0.6	6.2 ± 0.4	NR
^a^Vernon et al. 2003, USA [17]	16.8 ± NR	5.3 ± NR	NR
Secondary outcomes			
Bolus insulin (units/day)			
Buehler et al. 2021, USA [9]	33 ± NR	1 ± NR	NR
Eiswirth, Clark & Diamond, 2018, UK [10]	NR	NR	NR
Gardemann, Knowles & Marquardt, 2023, Germany [11]	20–24 ± NR	12.5 ± NR	NR
Kleiner et al. 2022, Italy [12]	18.3 ± 9.5	10.3 ± 6.5	< 0.001
Kwiendacz et al. 2019, Poland [13]	NR	14.4 ± NR	NR
^a^O’Neill et al. 2003, USA [14]	NR	NR	NR
Raab, 2003, Australia [15]	NR	NR	NR
Ranjan et al. 2017, Denmark [16]	16.3 ± 7.9	6.6 ± 1.8	0.001
^a^Vernon et al. 2003, USA [17]	NR	NR	NR
Weight (kg)			
Buehler et al. 2021, USA [9]	NR	NR	NR
Eiswirth, Clark & Diamond, 2018, UK [10]	NR	Decreased by 3	NR
Gardemann, Knowles & Marquardt, 2023, Germany [11]	61 ± NR	61 ± NR	NR
Kleiner et al. 2022, Italy [12]	68.9 ± 13.5	66.0 ± 6.8	NS
Kwiendacz et al. 2019, Poland [13]	NR	62 ± NR	NR
^a^O’Neill et al. 2003, USA [14]	NR	NR	NR
Raab, 2003, Australia [15]	84.0 ± NR	72 ± NR	NR
Ranjan et al. 2017, Denmark [16]	75.2 ± 11.7	72.9 ± 10.3	NR
^a^Vernon et al. 2003, USA [17]	61.2 ± NR	69.4 ± NR	NR
Quality of life (participant reported)			
Buehler et al. 2021, USA [9]	NR	NR	NR
Eiswirth, Clark & Diamond, 2018, UK [10]	NR	Improved	NR
Gardemann, Knowles & Marquardt, 2023, Germany [11]	NR	Improved	NR
Kleiner et al. 2022, Italy [12]	NR	NR	NR
Kwiendacz et al. 2019, Poland [13]	NR	NR	NR
^a^O’Neill et al. 2003, USA [14]	NR	NR	NR
Raab, 2003, Australia [15]	NR	Improved	NR
Ranjan et al. 2017, Denmark [16]	NR	NR	NR
^a^Vernon et al. 2003, USA [17]	NR	NR	NR

*HbA1c* glycated haemoglobin, *kg* kilograms, *NR* not reported, *NS* not significant, *T1D* type 1 diabetes, *T2D* type 2 diabetes

^a^Participants with T1D and T2D in this study – only T1D participant results are reported in this review

The Table 4 caption was inadvertently truncated as “Low carbohydrate diet (< 130 g/day or < 26% total energy intake) quantitative studies outcomes” but should read as “Low carbohydrate diet (< 130 g/day or < 26% total energy intake) quantitative studies outcomes quality of life section the reference for Turton et al. 2023, Australia reported as number [18]. Corrected to [22]”.

Incorrect Table 4 caption

**Table:** Low carbohydrate diet (< 130 g/day or < 26% total energy intake) quantitative studies outcomes

Author, year, country	Pre intervention	Post intervention	P-value
Primary outcome			
HbA1c (%)			
Krebs et al. 2016, New Zealand [18]	7.9 ± 0.9	7.2 ± 0.4	NS
^a^Ireland, O’Dea & Nankervis, 1992, Australia [19]	11.1 ± 0.6	11.6 ± 0.8	NS
Nielsen et al. 2012, Sweden [20]	7.6 ± 1.0	6.9 ± 1.0	< 0.001
Paul et al. 2022, Australia [21]	8.0 ± 1.7	7.1 ± 1.1	0.003
Schmidt et al. 2019, Denmark [7]	7.3 ± 0.5	7.4 ± 0.4	NS
Turton et al. 2023, Australia [22]	7.7 ± 0.5	7.1 ± 0.7	< 0.01
Secondary outcomes			
Bolus insulin (units/day)			
Krebs et al. 2016, New Zealand [18]	NR	NR	NR
^a^Ireland, O’Dea & Nankervis, 1992, Australia [19]	NR	NR	NR
Nielsen et al. 2012, Sweden [20]	NR	NR	NR
Paul et al. 2022, Australia [21]	20.3 ± 6.7	13.4 ± 6.5	< 0.0001
Schmidt et al. 2019, Denmark [7]	NR	15.1 ± 4.4	NR
Turton et al. 2023, Australia [22]	NR	NR	NR
Weight (kg)			
Krebs et al. 2016, New Zealand [18]	83.2 ± 11.0	78.0 ± 6.4	NS
^a^Ireland, O’Dea & Nankervis, 1992, Australia [19]	62.1 ± 3.1	61.9 ± 3.1	NS
Nielsen et al. 2012, Sweden [20]	77.6 ± 15	76.7 ± 14.6	NS
Paul et al. 2022, Australia [21]	79.3 ± 11.1	77.4 ± 11.3	0.013
Schmidt et al. 2019, Denmark [7]	77.4 ± 10.6	75.5 ± 10.9	0.012
Turton et al. 2023, Australia [22]	93.8 ± 18.7	91.4 ± 17.7	< 0.025
Quality of life (participant reported)			
Krebs et al. 2016, New Zealand [18]	NR	NR	NR
^a^Ireland, O’Dea & Nankervis, 1992, Australia [19]	NR	NR	NR
Nielsen et al. 2012, Sweden [20]	NR	NR	NR
Paul et al. 2022, Australia [21]	41.6 ± 11.2	40.5 ± 14.3	NS
Schmidt et al. 2019, Denmark [7]	30.9 ± 3.8	27.1 ± 6.5	NS
Turton et al. 2023, Australia [22]	33.8 ± 5.8	30.3 ± 7.4	< 0.025

*HbA1c* glycated haemoglobin, *kg* kilograms, *NR* not reported, *NS* not significant

^a^This study contained two interventions. One intervention used a low fat, low carbohydrate diet and the other used a high fat, low carbohydrate diet. The high fat, low carbohydrate diet intervention did not meet the definition of a low carbohydrate diet (< 130 g/day or < 26% total energy intake) and was excluded from this review. The low fat, low carbohydrate diet intervention did meet the definition of a low carbohydrate diet and was therefore included in this review [19]

Correct Table 4 caption

**Table 4:** Low carbohydrate diet (< 130 g/day or < 26% total energy intake) quantitative studies outcomes quality of life section the reference for Turton et al. 2023, Australia reported as number [18]. Corrected to [22]

Author, year, country	Pre intervention	Post intervention	P-value
Primary outcome			
HbA1c (%)			
Krebs et al. 2016, New Zealand [18]	7.9 ± 0.9	7.2 ± 0.4	NS
^a^Ireland, O’Dea & Nankervis, 1992, Australia [19]	11.1 ± 0.6	11.6 ± 0.8	NS
Nielsen et al. 2012, Sweden [20]	7.6 ± 1.0	6.9 ± 1.0	< 0.001
Paul et al. 2022, Australia [21]	8.0 ± 1.7	7.1 ± 1.1	0.003
Schmidt et al. 2019, Denmark [7]	7.3 ± 0.5	7.4 ± 0.4	NS
Turton et al. 2023, Australia [22]	7.7 ± 0.5	7.1 ± 0.7	< 0.01
Secondary outcomes			
Bolus insulin (units/day)			
Krebs et al. 2016, New Zealand [18]	NR	NR	NR
^a^Ireland, O’Dea & Nankervis, 1992, Australia [19]	NR	NR	NR
Nielsen et al. 2012, Sweden [20]	NR	NR	NR
Paul et al. 2022, Australia [21]	20.3 ± 6.7	13.4 ± 6.5	< 0.0001
Schmidt et al. 2019, Denmark [7]	NR	15.1 ± 4.4	NR
Turton et al. 2023, Australia [22]	NR	NR	NR
Weight (kg)			
Krebs et al. 2016, New Zealand [18]	83.2 ± 11.0	78.0 ± 6.4	NS
^a^Ireland, O’Dea & Nankervis, 1992, Australia [19]	62.1 ± 3.1	61.9 ± 3.1	NS
Nielsen et al. 2012, Sweden [20]	77.6 ± 15	76.7 ± 14.6	NS
Paul et al. 2022, Australia [21]	79.3 ± 11.1	77.4 ± 11.3	0.013
Schmidt et al. 2019, Denmark [7]	77.4 ± 10.6	75.5 ± 10.9	0.012
Turton et al. 2023, Australia [22]	93.8 ± 18.7	91.4 ± 17.7	< 0.025
Quality of life (participant reported)			
Krebs et al. 2016, New Zealand [18]	NR	NR	NR
^a^Ireland, O’Dea & Nankervis, 1992, Australia [19]	NR	NR	NR
Nielsen et al. 2012, Sweden [20]	NR	NR	NR
Paul et al. 2022, Australia [21]	41.6 ± 11.2	40.5 ± 14.3	NS
Schmidt et al. 2019, Denmark [7]	30.9 ± 3.8	27.1 ± 6.5	NS
Turton et al. 2023, Australia [22]	33.8 ± 5.8	30.3 ± 7.4	< 0.025

*HbA1c* glycated haemoglobin, *kg* kilograms, *NR* not reported, *NS* not significant

^a^This study contained two interventions. One intervention used a low fat, low carbohydrate diet and the other used a high fat, low carbohydrate diet. The high fat, low carbohydrate diet intervention did not meet the definition of a low carbohydrate diet (< 130 g/day or < 26% total energy intake) and was excluded from this review. The low fat, low carbohydrate diet intervention did meet the definition of a low carbohydrate diet and was therefore included in this review [19]
