# Supplementary material for: Low carbohydrate diets, glycaemic control, enablers, and barriers in the management of type 1 diabetes: a mixed methods systematic review
Source: Diabetol Metab Syndr. 2024 Nov 2;16:261. doi: 10.1186/s13098-024-01496-5 (PMC11531154; doi:10.1186/s13098-024-01496-5)
Supplement: Supplementary file 2 — Additional file 2: MEDLINE (Ovid) search strategy for included studies (1946 to 1 October 2023). [file 13098_2024_1496_MOESM2_ESM.docx]

**Additional file 2** MEDLINE (Ovid) search strategy for included studies (1946 to 1 October 2023)

***Quantitative studies***

| **Search ID number** | **Search terms** |
| --- | --- |
| 1 | diabetes mellitus, type 1/ |
| 2 | (diabet* adj5 "type 1").tw. |
| 3 | dmt1.tw. |
| 4 | t1dm.tw. |
| 5 | ("insulin dependent" adj5 diabet*).tw. |
| 6 | or/1-5 |
| 7 | diet, carbohydrate-restricted/ or diet, ketogenic/ |
| 8 | ((low or restrict* or modif* or limit* or reduc*) adj4 carb*).tw. |
| 9 | (diet* adj4 modif*).tw. |
| 10 | (carb* adj4 diet*).tw |
| 11 | (keto* adj4 diet*).tw. |
| 12 | (interven* and carbohydrat* and diabet*).tw. |
| 13 | (diabet* and carbohydrat* and carbohydrat* diet*).tw. |
| 14 | or/7-13 |
| 15 | 6 and 14 |
| 16 | limit 15 to English language |

***Qualitative studies***

| **Search ID number** | **Search terms** |
| --- | --- |
| 1 | diabetes mellitus, type 1/ |
| 2 | (diabet* adj5 "type 1").tw. |
| 3 | dmt1.tw. |
| 4 | t1dm.tw. |
| 5 | ("insulin dependent" adj5 diabet*).tw. |
| 6 | or/1-5 |
| 7 | diet, carbohydrate-restricted/ or diet, ketogenic/ |
| 8 | ((low or restrict* or modif* or limit* or reduc*) adj4 carb*).tw. |
| 9 | (diet* adj4 modif*).tw. |
| 10 | (carb* adj4 diet*).tw |
| 11 | (keto* adj4 diet*).tw. |
| 12 | (interven* and carbohydrat* and diabet*).tw. |
| 13 | (diabet* and carbohydrat* and carbohydrat* diet*).tw. |
| 14 | or/7-13 |
| 15 | 6 and 14 |
| 16 | qualitative research/ |
| 17 | "Surveys and Questionnaires"/ |
| 18 | Focus Groups/ |
| 19 | grounded theory/ |
| 20 | qualitativ*.tw. |
| 21 | (survey* or interview* or questionnair* or focus group*).tw. |
| 22 | personal narrative/ |
| 23 | narrativ*.tw. |
| 24 | "reproducibility of results"/ |
| 25 | (valid* and reliab*).tw. |
| 26 | ("action research" or "document analysis" or ethnograph* or ethnologic* or "grounded theory" or naturalistic or phenomenol*).tw. |
| 27 | or/16-26 |
| 28 | 15 and 27 |
| 29 | limit 15 to English language |
