# Supplementary material for: Low carbohydrate diets, glycaemic control, enablers, and barriers in the management of type 1 diabetes: a mixed methods systematic review
Source: Diabetol Metab Syndr. 2024 Nov 2;16:261. doi: 10.1186/s13098-024-01496-5 (PMC11531154; doi:10.1186/s13098-024-01496-5)
Supplement: Supplementary file 3 — Additional file 3: Inclusion and exclusion criteria for selection of quantitative and qualitative studies. [file 13098_2024_1496_MOESM3_ESM.docx]

**Additional file 3** Inclusion and exclusion criteria for selection of quantitative and qualitative studies

| **Parameter** | **Inclusion criteria** | **Exclusion criteria** |
| --- | --- | --- |
| Population | For quantitative and qualitative studies:   - Adults (≥18 years) with T1D. - Males, females, unspecified gender. - Diagnosis of T1D ≥one year. - All ethnicities. | For quantitative and qualitative studies:   - Aged ≤17 years. - Participants with T2D. - Participants with gestational diabetes. - Breast-feeding participants. - Animal studies. |
| Phenomenon of interest | For quantitative studies:   - Studies that used a very low (≤50g/day or <10% TEI) or low carbohydrate dietary intake (<130g/day or <26% TEI).   For qualitative studies:   - Studies that reported participants’ perceptions, knowledge, and experiences with using a very low (≤50g/day or <10%% TEI) or a low carbohydrate dietary intake (<130g/day or <26% TEI). | For quantitative studies:   - Studies that used a dietary carbohydrate intake of ≥130g/day or ≥26% TEI.   For qualitative studies:   - Studies that did not report participants’ perceptions, knowledge, and experiences and/or used a dietary carbohydrate intake of ≥130g/day or ≥26% TEI. |
| Context | For quantitative and qualitative studies:   - Studies that were conducted in the participants’ natural setting. | For quantitative and qualitative studies:   - Studies conducted in an inpatient hospital setting. |
| Study design | For quantitative studies:   - Only primary studies. - All study designs (i.e. experimental/interventional and non-experimental/observational) regardless of study duration or sample size. - Studies that reported changes in glycaemic control i.e. HbA1c (pre vs. post intervention). - Studies published in English.   For qualitative studies:   - Qualitative studies using descriptive approaches, case studies, ethnography, phenomenology, grounded theory, narrative inquiry, participatory methodologies, and the qualitative component of a mixed methods study. - Studies published in English. | For quantitative studies:   - Studies where both T1D and T2D and children/adolescents and adults were included but the results of each group were not clearly reported. - Studies that only reported pre or post HbA1c. - Non-English studies.   For qualitative studies:   - Commentaries, conference abstracts/proceedings, secondary studies (such as reviews and meta-analysis), editorials, books, discussion papers, thesis, diagnostic studies, and grey literature. - Studies where both T1D and T2D and children/adolescents and adults were included but the results of each group were not clearly reported. - Non-English studies. |

**Legend: *g* grams, *HbA1c* glycated haemoglobin, *TEI* total energy intake, *T1D* type 1 diabetes, *T2D* type 2 diabetes.**
