# Supplementary material for: Low carbohydrate diets, glycaemic control, enablers, and barriers in the management of type 1 diabetes: a mixed methods systematic review
Source: Diabetol Metab Syndr. 2024 Nov 2;16:261. doi: 10.1186/s13098-024-01496-5 (PMC11531154; doi:10.1186/s13098-024-01496-5)
Supplement: Supplementary file 4 — Additional file 4: Key variables extracted from included quantitative and qualitative studies. [file 13098_2024_1496_MOESM4_ESM.docx]

**Additional file 4** Key variables extracted from quantitative and qualitative included studies

| **Quantitative studies**  **Key variables extracted** | **Details** |
| --- | --- |
| Author/year/country of origin | Author name.  Publication year.  UK, USA, Australia, Europe, New Zealand. |
| Study characteristics | Study design (RCT, quasi-experimental, case series, case reports, mixed methods).  Number of participants.  Dietary intervention duration.  Macronutrient distribution of the intervention dietary intervention.  Form of dietary support provided to participants. |
| Participant characteristics | Age.  Diabetes duration.  Baseline HbA1c. |
| Primary outcome | HbA1c. |
| Secondary outcomes | Bolus insulin, weight, quality of life, and dietary adherence. |
| **Qualitative studies**  **Key variables extracted** | **Details** |
| Author/year/country of origin | Author name.  Publication year.  Canada, Australia, New Zealand. |
| Research aim/objectives | Research aim/objectives of each study. |
| Participant characteristics | Age (if available).  Diabetes duration (if available).  Baseline HbA1c (if available). |
| Study characteristics | Qualitative design, mixed methods.  Number of study participants.  Study setting: participants in their ‘natural’ environment. |
| Intervention duration/  year of data collection | Length of the study: March to May 2013 vs 12-week duration.  Year data was collected: pre vs post study vs only post intervention. |
| Key findings/conclusions | Key findings/conclusions of each study. |

**Legend: *g* grams,** ***HbA1c* glycated haemoglobin**
