# Supplementary material for: Low carbohydrate diets, glycaemic control, enablers, and barriers in the management of type 1 diabetes: a mixed methods systematic review
Source: Diabetol Metab Syndr. 2024 Nov 2;16:261. doi: 10.1186/s13098-024-01496-5 (PMC11531154; doi:10.1186/s13098-024-01496-5)
Supplement: Supplementary file 5 — Additional file 5: Intervention calories, macronutrient distribution, and dietary support methods of included quantitative studies. [file 13098_2024_1496_MOESM5_ESM.docx]

**Additional file 5** Quality appraisal of studies^a^

| **Author, Year, Country** | **Study design** | **Q1** | **Q2** | **Q3** | **Q4** | **Q5** | **Q6** | **Q7** | **Q8** | **Q9** | **Q10** | **Q11** | **Q12** | **Total** |
| --- | --- | --- | --- | --- | --- | --- | --- | --- | --- | --- | --- | --- | --- | --- |
| Buehler, 2021, UK [9] | Case report  (retrospective) | Y | Y | Y | Y | Y | Y | Y | Y | X | X | X | X | 8/8 |
| Eiswirth, Clark, & Diamond, 2018, UK [10] | Case report  (retrospective) | N | Y | Y | Y | Y | Y | Y | Y | X | X | X | X | 7/8 |
| Gardemann, Knowles, & Marquardt, 2023, Germany [11] | Case report  (retrospective) | N | Y | Y | Y | Y | Y | Y | U | X | X | X | X | 6½/8 |
| Kleiner et al. 2022, Italy [12] | Quasi-experimental | Y | Y | N | N | Y | Y | U | Y | Y | X | X | X | 6½/9 |
| Kwiendacz et al. 2019, Poland [13] | Case report  (retrospective) | Y | Y | Y | Y | Y | Y | U | U | X | X | X | X | 7/8 |
| O’Neill et al. 2003, USA [14] | Case series  (retrospective) | Y | Y | Y | U | U | Y | Y | Y | Y | Y | X | X | 9/10 |
| Raab, 2003, Australia [15] | Case report  (retrospective) | N | Y | Y | Y | Y | Y | Y | Y | X | X | X | X | 7/8 |
| Ranjan et al. 2017, Denmark [16] | RCT crossover | U | U | Y | N | N | Y | U | Y | U | Y | Y | Y | 8/12 |
| Vernon et al. 2003, USA [17] | Case series  (retrospective) | Y | Y | Y | U | U | Y | Y | Y | Y | Y | X | X | 9/10 |
| Krebs et al. 2016, New Zealand [18] | RCT  parallel | Y | Y | Y | N | N | Y | N | Y | Y | Y | Y | Y | 9/12 |
| ^b^Ireland, O’Dea, & Nankervis, 1992, Australia [19] | Quasi-experimental | Y | Y | Y | N | Y | Y | U | Y | Y | X | X | X | 7½/9 |
| Nielsen et al. 2012, Sweden [20] | Quasi-experimental | Y | Y | N | N | Y | Y | U | Y | Y | X | X | X | 6½/9 |
| Paul et al. 2022, Australia [21] | Quasi-experimental Quantitative component of a mixed methods study | Y | Y | N | N | Y | Y | U | Y | Y | X | X | X | 6½/9 |
| Schmidt et al. 2019, Denmark [7] | RCT crossover | U | U | Y | N | N | Y | U | Y | U | Y | Y | Y | 8/12 |
| Turton et al. 2023, Australia [22] | Case series  (retrospective) | Y | Y | Y | Y | Y | Y | Y | Y | Y | Y | X | X | 10/10 |
| Cresswell et al. 2015, New Zealand [49] | Qualitative | U | Y | Y | Y | Y | N | N | Y | Y | Y | X | X | 7½/10 |
| Paul et al. 2022, Australia [21] | Qualitative component of a mixed methods study | N | Y | Y | Y | Y | N | N | Y | Y | Y | X | X | 7/10 |
| Wong et al. 2021, Canada [48] | Qualitative | U | Y | Y | Y | Y | N | U | Y | U | Y | X | X | 7½/10 |

**Legend: *Y* yes, *N* no, *U* unclear, *N/A* Not appliable, *RCT* randomised control trial, *Q* question number, *X* no question in this checklist.**

**Question scores: Y= 1, N= 0, U= 1/2, N/A= 0**

**^a^All included studies were appraised using the appropriate critical appraisal checklist sourced from the Joanna Briggs Institute. Quantitative studies were appraised using the appropriate appraisal checklist according to the study design. The mixed methods study components were separately appraised using quantitative and qualitative appraisal checklists. Qualitative studies were appraised using the critical appraisal checklist for qualitative research.**

**^b^This study contained two interventions. One intervention used a** **low fat, low carbohydrate diet and the other used a high fat, low carbohydrate diet. The high fat, low carbohydrate diet intervention did not meet the definition of a low carbohydrate diet (<130g/day or <26% total energy intake) and was excluded from this review. The low fat, low carbohydrate diet intervention did meet the definition of a low carbohydrate diet and was therefore included in this review [19].**
