# Supplementary material for: Low carbohydrate diets, glycaemic control, enablers, and barriers in the management of type 1 diabetes: a mixed methods systematic review
Source: Diabetol Metab Syndr. 2024 Nov 2;16:261. doi: 10.1186/s13098-024-01496-5 (PMC11531154; doi:10.1186/s13098-024-01496-5)
Supplement: Supplementary file 6 — Additional file 6: Quality appraisal of studies. [file 13098_2024_1496_MOESM6_ESM.docx]

**Additional file 6** Intervention calories, macronutrient distribution, and dietary support methods of included quantitative studies

| **Very low carbohydrate diet (≤50g/day or <10% of total energy intake)** | | | | | |  |
| --- | --- | --- | --- | --- | --- | --- |
| **Author, Year,**  **Country** | **Intervention vs.**  **comparison diet** | **Calories**  **(per day)** | **CHO**  **(per day)** | **Protein**  **(per day)** | **Fat**  **(per day)** | **Dietary support** |
| Buehler, 2021, UK [9] | <30g/day CHO vs. no comparison | NR | <30g | NR | NR | In person |
| Eiswirth, Clark, & Diamond, 2018, UK [10] | 30-50g/day CHO vs. no comparison | NR | 30-50g | NR | NR | NR |
| Gardemann, Knowles, & Marquardt, 2023, Germany [11] | 24-30g/day CHO vs. 140g/day CHO | NR | 24-30g | NR | NR | NR |
| Kleiner et al. 2022, Italy [12] | <50g/day CHO vs >200g/day CHO | NR | 5% | 25% | 70% | NR |
| Kwiendacz et al. 2019, Poland [13] | 10g/day CHO vs. no comparison | NR | 10g | 15g | 120g | NR |
| ^a^O’Neill et al. 2003, USA [14] | 30g/day CHO vs. no comparison | NR | 30g | NR | NR | Education |
| Raab, 2003, Australia [15] | 30-50g/day CHO vs. no comparison | NR | 30-50g | NR | NR | NR |
| Ranjan et al. 2017, Denmark [16] | ≤50g/day CHO vs. ≥250g/day CHO | 1,876±278 | 47±10g | 143±3g | 124±1.8g | Meal plan |
| ^a^Vernon et al. 2003, USA [17] | <20g/day vs. no comparison | NR | <20g | NR | NR | Education |
| **Low carbohydrate diet** **(<130g/day** **or <26% of total energy intake)** | | | | | |  |
| **Author, Year** | **Intervention vs. comparison diet** | **Calories**  **(per day)** | **CHO**  **(per day)** | **Protein**  **(per day)** | **Fat**  **(per day)** | **Intervention support** |
| Krebs et al. 2016, New Zealand [18] | 50-70g/day CHO vs. CHO counting | 1391±159 | 103±22g | 76±16g | 69±13g | Education |
| **^b^**Ireland, O’Dea, & Nankervis, 1992, Australia [19] | LF/LC diet vs. self-selected diet | 1,617±109 | 87±5g | 250g | 28g | Education |
| Nielsen et al. 2012, Sweden [20] | ≤75g/day CHO vs. no comparison | NR | ≤75g | NR | NR | Education |
| Paul et al. 2022, Australia [21] | <100g/day CHO vs. no comparison | 1,110±279 | 69±24g | 83g±24 | 50±20g | Education, meal plan |
| Schmidt et al. 2019, Denmark [7] | <100g/day CHO vs. >250g/day CHO | NR | 98±11g | NR | NR | Education, meal plan |
| Turton et al. 2023, Australia [22] | 25-75g/day CHO vs >150g/day CHO | 1,939±629 | 63±51g | 118±28g | 130±57g | Education, sample meal plans |

**Legend: *CHO* carbohydrate, *g* grams, *LC* low carbohydrate, *LF* low fat, *NR* not reported, *T1D*** **type 1 diabetes, *T2D* type 2 diabetes, *vs* versus.**

**This table shows the calories and macronutrient amounts consumed by the study group.**

**^a^Participants with T1D and T2D in this study – only T1D participant results are reported in this review.**

**^b^This study contained two interventions. One intervention used a** **low fat, low carbohydrate diet and the other used a high fat, low carbohydrate diet. The high fat, low carbohydrate diet intervention did not meet the definition of a low carbohydrate diet (<130g/day or <26% total energy intake) and was excluded from this review. The low fat, low carbohydrate diet intervention did meet the definition of a low carbohydrate diet and was therefore included in this review [19].**
